# Supplementary material for: Complexome profiling of the Chlamydomonas psb28 mutant reveals TEF5 as an early PSII assembly factor
Source: Plant Cell. 2025 Mar 17;37(6):koaf055. doi: 10.1093/plcell/koaf055 (PMC12164590; doi:10.1093/plcell/koaf055)
Supplement: koaf055_Supplementary_Data [file koaf055_supplementary_data.zip › psb28_interactive.zip!/plots/intensity/qualityPlots/peptides/CCB4_Cre08.g382300.t1.2.html]

### Cre08.g382300.t1.2

Trivial name: CCB4  
SeqCoverage [median]: 12.70  
Mass: 32.23 kDa  
  
 

| Sequence | MissedCleavage | Mass [Da] | wtIntensity | muIntensity | Score |
| --- | --- | --- | --- | --- | --- |
| ALSGIAPVVDASSSQSR | 0 | 1643.85 | 1458.19 | 5886.41 | 91.86 |
| DGVIVAGTDTVR | 0 | 1201.63 | 0.00 | 4937.27 | 121.80 |
| TLFHYGFITK | 0 | 1225.65 | 706.47 | 2051.19 | 88.68 |
|  |  |  | 2164.7 | 12874.9 |  |
